# Supplementary material for: The Ambiguous Correlation of Blautia with Obesity: A Systematic Review
Source: Microorganisms. 2024 Aug 26;12(9):1768. doi: 10.3390/microorganisms12091768 (PMC11433710; doi:10.3390/microorganisms12091768)
Supplement: Supplementary file 1 [file microorganisms-12-01768-s001.zip › microorganisms-3157637-supplementary.pdf]

Table S1. A list of *Blautia* species validly and correctively nominated under the International Code of Nomenclature of Prokaryotes (bacteriological code). Note that the genus *Blautia* is comprised of 53 child taxa as of 18 July 2024 (https://lpsn.dsmz.de/genus/blautia, accessed on 18 July 2024).

	Species Name	Brief Description	Refs.
1	*B. acetigignens*	• acetic acid-producing species<br>• isolated from human feces<br>• DNA G+C content is 44.4%<br>• Type strain: DSM 102165; JCM 34803; Sanger_28	Hitch et al. 2022 [1]
2	*B. ammoniilytica*	• ammonia-degrading species<br>• isolated from human feces<br>• DNA G+C content is 44.4%<br>• Type strain: DSM 102163; JCM 34802; Sanger_23	Hitch et al. 2022 [1]
3	*B. argi*	• produces acetic and lactic acids<br>• isolated from dog feces<br>• DNA G+C content is 44.2%<br>• Type strain: JCM 31394; KCTC 15426; N6H1-15	Paek et al. 2019 [2]
4	*B. caecimuris*	• produces acid<br>• Isolated from a caecum of mouse<br>• G+C content is 43.0%<br>• Type strain: DSM 29492; KCTC 15541; SJ18	Lagkouvardos et al. 2016 [3]
5	*B. celeris*	• fast growing species<br>• isolated from human feces<br>• DNA G+C content is 54.21%<br>• Type strain: CGMCC 1.32807; KCTC 25086; NSJ 34	Liu et al. 2022 [4]
6	*B. coccoides*	• grape-like shape and produces succinic acid<br>• isolated from mice feces<br>• DNA G+C content is 43–45%<br>• Type strain: ATCC 29236; DSM 935; JCM 1395; NCTC 11035	(Kaneuchi et al. 1976) [5] Liu et al. 2008 [6]
7	*B. faecicola*	• produces acetic acid<br>• isolate from human feces<br>• DNA G+C content is 44.7%<br>• Type strain: DSM 107827; KCTC 15706; KGMB01111	Kim et al. 2020 [7]
8	*B. faecis*	• Produces lactic and acetic acids<br>• Isolated from human feces<br>• DNA G+C content is 41.6%<br>• Type strain: DSM 27629; JCM 17205; KCTC 5980; M25	Park et al. 2013 [8]
9	*B. fusiformis*	• spindle shaped species<br>• isolated from human feces<br>• DNA G+C content is 44.1%<br>• Type strain: CLA-AA-H217; DSM 112726; JCM 35880	Afrizal et al. 2023 [9]
10	*B. glucerasea*	• able to hydrolyze glucosylceramide and produces acetate, formate, and lactate<br>• isolated from dog feces<br>• DNA G+C content is 40.7%<br>• Type strain: DSM 22028; HFTH-1; NBRC 104932	Furuya et al. 2010 [10]
11	*B. hansenii*	• named after P. Arne Hansen, a Danish-American bacteriologist, and isolated from human feces<br>• DNA G+C content is 41.1%<br>• Type strain: ATCC 27752; CIP 104219; DSM 20583; JCM 14655	(Holdeman and Moore 1974) [11] Liu et al. 2008 [6]
12	*B. hominis*	• produces acetic acid, succinic acid, lactic acid and fumaric acid<br>• isolated from human feces	Shin et al. 2018 [12]

		• DNA G+C content is 46.3%<br>• Type strain: JCM 32276; KB1; KCTC 15618	
13	*B. hydrogenotrophica*	• able to use H_2/CO_2 as energy source and produces acetic acid<br>• isolated from human feces<br>• DNA G+C content is 45.2%<br>• Type strain: DSM 10507; JCM 14656; S5a33; S5a36	(Bernalier et al. 1997) [13]<br>Liu et al. 2008 [6]
14	*B. intestinalis*	• Produces butyric acid<br>• Isolated from human feces<br>• DNA G+C content is 42.36%<br>• Type strain: 27–44; CGMCC 1.5285; NBRC 113774	Wang et al. 2021 [14]
15	*B. liquoris*	• produces acetic acid<br>• isolated from the mud in a fermentation cellar used for the production of Chinese strong-flavor liquor<br>• DNA G+C content is 42.1%<br>• Type strain: CGMCC 1.5299; CGMCC T 1.5299; JCM 34225; KCTC 25163; LZLJ-3	Lu et al. 2021 [15]
16	*B. luti*	• produces acetate, succinate, and hydrogen<br>• isolated from human feces<br>• DNA G+C content is 43.3%<br>• Type strain: BInIX; CCUG 45635; DSM 14534	(Simmering et al. 2002) [16]<br>Liu et al. 2008 [6]
17	*B. obeum*	• Oval shaped species and produces acetic acid and ethanol<br>• Isolated from human feces<br>• DNA G+C content is 41%<br>• Type strain: ATCC 29174; DSM 25238; KCTC 15206	(Moore et al. 1976) [17]<br>Lawson and Finegold 2015 [18]
18	*B. parvula*	• Species with small or little cells, and produces acetic acid, lactic acid and succinic acid<br>• isolated from human feces<br>• DNA G+C content is 46.7%<br>• Type strain: BCRC 81349; DN0138; NBRC 113351	Miura et al. 2023 [19]
19	*B. producta*	• Produces acetic and hydrogen<br>• Isolated from human feces<br>• DNA G+C content is 45%<br>• Type strain: ATCC 27340; CCUG 10976; CCUG 9990; DSM 2950; JCM 1471	(Prévot 1941)<br>Liu et al. 2008 [6]
20	*B. pseudococcoides*	• falsely recognized as *Blautia coccoides*<br>• Type strain: DSM 26115; JCM 35243; YL58	Maturana and Cárdenas 2022 [20]
21	*B. schinkii*	• named after Bernard Schink, produces acetic acid<br>• isolated from rumen content of suckling lamb<br>• DNA G+C content is 46.4%<br>• Type strain: Bie 41; CCUG 53897; CIP 105464; DSM 10518; JCM 14657	(Rieu-Lesme et al. 1997) [21]<br>Liu et al. 2008 [6]
22	*B. stercoris*	• Produces acetic acid<br>• Isolated from human feces<br>• DNA G+C content is 35.6%<br>• Type strain: GAM 6-1; JCM 17204; KCTC 5981	Park et al. 2012 [22]
23	*B. wexlerae*	• named in honor of the American microbiologist Hannah M. Wexler<br>• isolated from huma feces<br>• Type strain: ATCC BAA-1564; DSM 19850; WAL 14507	Liu et al. 2008 [6]

Reference

1. Hitch TCA, Riedel T, Oren A, Overmann J, Lawley TD, Clavel T: **Automated analysis of genomic sequences facilitates high-throughput and comprehensive description of bacteria**. *ISME Communications* 2021, **1**(1):16.
2. Paek J, Shin Y, Kook JK, Chang YH: **Blautia argi sp. nov., a new anaerobic bacterium isolated from dog faeces**. *Int J Syst Evol Microbiol* 2019, **69**(1):33-38.
3. Lagkouvardos I, Pukall R, Abt B, Foesel BU, Meier-Kolthoff JP, Kumar N, Bresciani A, Martínez I, Just S, Ziegler C *et al*: **The Mouse Intestinal Bacterial Collection (miBC) provides host-specific insight into cultured diversity and functional potential of the gut microbiota**. *Nat Microbiol* 2016, **1**(10):16131.
4. Liu C, Du MX, Abuduaini R, Yu HY, Li DH, Wang YJ, Zhou N, Jiang MZ, Niu PX, Han SS *et al*: **Correction: Enlightening the taxonomy darkness of human gut microbiomes with a cultured biobank**. *Microbiome* 2022, **10**(1):163.
5. Kaneuchi C, Benno Y, Mitsuoka T: **Clostridium coccoides, a New Species from the Feces of Mice**. *International Journal of Systematic and Evolutionary Microbiology* 1976, **26**(4):482-486.
6. Liu C, Finegold SM, Song Y, Lawson PA: **Reclassification of Clostridium coccoides, Ruminococcus hansenii, Ruminococcus hydrogenotrophicus, Ruminococcus luti, Ruminococcus productus and Ruminococcus schinkii as Blautia coccoides gen. nov., comb. nov., Blautia hansenii comb. nov., Blautia hydrogenotrophica comb. nov., Blautia luti comb. nov., Blautia producta comb. nov., Blautia schinkii comb. nov. and description of Blautia wexlerae sp. nov., isolated from human faeces**. *Int J Syst Evol Microbiol* 2008, **58**(Pt 8):1896-1902.
7. Kim J-S, Park J-E, Lee KC, Choi S-H, Oh BS, Yu SY, Eom MK, Kang SW, Han K-I, Suh MK *et al*: **Blautia faecicola sp. nov., isolated from faeces from a healthy human**. *International Journal of Systematic and Evolutionary Microbiology* 2020, **70**(3):2059-2065.
8. Park SK, Kim MS, Bae JW: **Blautia faecis sp. nov., isolated from human faeces**. *Int J Syst Evol Microbiol* 2013, **63**(Pt 2):599-603.
9. Afrizal A, Hitch TCA, Viehof A, Treichel N, Riedel T, Abt B, Buhl EM, Kohlheyer D, Overmann J, Clavel T: **Anaerobic single-cell dispensing facilitates the cultivation of human gut bacteria**. *Environ Microbiol* 2022, **24**(9):3861-3881.
10. Furuya H, Ide Y, Hamamoto M, Asanuma N, Hino T: **Isolation of a novel bacterium, Blautia glucerasei sp. nov., hydrolyzing plant glucosylceramide to ceramide**. *Arch Microbiol* 2010, **192**(5):365-372.
11. Holdeman LV, Moore WEC: **New Genus, Coprococcus, Twelve New Species, and Emended Descriptions of Four Previously Described Species of Bacteria from Human Feces**. *International Journal of Systematic and Evolutionary Microbiology* 1974, **24**(2):260-277.
12. Shin NR, Kang W, Tak EJ, Hyun DW, Kim PS, Kim HS, Lee JY, Sung H, Whon TW, Bae JW: **Blautia hominis sp. nov., isolated from human faeces**. *Int J Syst Evol Microbiol* 2018, **68**(4):1059-1064.
13. Bernalier A, Willems A, Leclerc M, Rochet V, Collins MD: **Ruminococcus hydrogenotrophicus sp. nov., a new H2/CO2-utilizing acetogenic bacterium isolated from human feces**. *Arch Microbiol* 1996, **166**(3):176-183.
14. Wang YJ, Abdugheni R, Liu C, Zhou N, You X, Liu SJ: **Blautia intestinalis sp. nov., isolated from human feces**. *Int J Syst Evol Microbiol* 2021, **71**(9).
15. Lu LF, Yang Y, Chai LJ, Lu ZM, Zhang LQ, Qin H, Yang P, Xu ZH, Shen CH: **Blautia liquoris sp. nov., isolated from the mud in a fermentation cellar used for the production of Chinese strong-flavour liquor**. *Int J Syst Evol Microbiol* 2021, **71**(10).

16. Simmering R, Taras D, Schwiertz A, Le Blay G, Gruhl B, Lawson PA, Collins MD, Blaut M: **Ruminococcus luti sp. nov., isolated from a human faecal sample**. *Syst Appl Microbiol* 2002, **25**(2):189-193.
17. MOORE WEC, JOHNSON JL, HOLDEMAN LV: **Emendation of Bacteroidaceae and Butyrivibrio and Descriptions of Desulfomonas gen. nov. and Ten New Species in the Genera Desulfomonas, Butyrivibrio, Eubacterium, Clostridium, and Ruminococcus**. *International Journal of Systematic and Evolutionary Microbiology* 1976, **26**(2):238-252.
18. Lawson PA, Finegold SM: **Reclassification of Ruminococcus obeum as Blautia obeum comb. nov**. *Int J Syst Evol Microbiol* 2015, **65**(Pt 3):789-793.
19. Miura T, Shimamura M, Yamazoe A, Kawasaki H: **Blautia parvula sp. nov., isolated from Japanese faecal samples**. *Int J Syst Evol Microbiol* 2023, **73**(11).
20. Maturana JL, Cárdenas JP: **Insights on the Evolutionary Genomics of the Blautia Genus: Potential New Species and Genetic Content Among Lineages**. *Front Microbiol* 2021, **12**:660920.
21. Rieu-Lesme F, Morvan B, Collins MD, Fonty G, Willems A: **A new H2/CO2-using acetogenic bacterium from the rumen: description of Ruminococcus schinkii sp. nov**. *FEMS Microbiol Lett* 1996, **140**(2-3):281-286.
22. Park SK, Kim MS, Roh SW, Bae JW: **Blautia stercoris sp. nov., isolated from human faeces**. *Int J Syst Evol Microbiol* 2012, **62**(Pt 4):776-779.
